# MicroRNA biogenesis pathway alterations in aging

**DOI:** 10.20517/evcna.2023.29

**Published:** 2023-08-18

**Authors:** Jorge Sanz-Ros, Cristina Mas-Bargues, Nekane Romero-García, Javier Huete-Acevedo, Mar Dromant, Consuelo Borrás

**Affiliations:** ^1^Freshage Research Group, Department of Physiology, Faculty of Medicine, University of Valencia, Centro de Investigación Biomédica en Red Fragilidad y Envejecimiento Saludable-Instituto de Salud Carlos III (CIBERFES-ISCIII), INCLIVA, Valencia 46010, Spain.; ^2^Department of Cardiology, Hospital Universitari i Politècnic La Fe, Valencia 46026, Spain.; ^3^Department of Anesthesiology and Surgical Trauma Intensive Care, Hospital Clinic Universitari de Valencia, University of Valencia, Valencia 46010, Spain.

**Keywords:** MicroRNA biogenesis, aging process, lifespan, intercellular communication

## Abstract

Aging is characterized by genomic instability and dysregulation of gene expression. MicroRNAs (miRNAs) are small non-coding RNAs that play a crucial role in post-transcriptional gene regulation. This work explores the impact of dysregulated miRNA biogenesis on the aging process. During aging, alterations in the transcription of primary miRNAs (pri-miRNAs) occur due to genomic changes, DNA damage, and epigenetic modifications. The microprocessor complex, comprising DGCR8 and Drosha proteins, is vital for pri-miRNA processing. Age-related changes in this complex affect miRNA biogenesis and miRNA expression profiles, linking these alterations with age-related conditions. Conversely, interventions like caloric restriction and mTOR inhibition enhance microprocessor activity, suggesting a connection between microprocessor function, aging-related pathways, and lifespan extension. Exportin-5 mediates the transport of pre-miRNAs from the nucleus to the cytoplasm. Although the role of miRNA export in aging is not well understood, accelerated export of pre-miRNAs is observed in response to DNA damage, and nucleocytoplasmic transport has been linked to cellular senescence. Dicer is responsible for processing pre-miRNAs into mature miRNAs. Reduced Dicer expression during aging is reported in various organisms and tissues and is associated with premature aging phenotypes. Conversely, the upregulation of Dicer improves stress resistance and metabolic adaptations induced by caloric restriction and exercise training. Understanding the role of miRNA biogenesis disruption in aging provides insights into the molecular mechanisms of aging and age-related diseases. Targeting this pathway may hold promise for therapeutic strategies and contribute to healthy aging.

## INTRODUCTION

During organismal aging, cells and tissues experience various changes that ultimately contribute to the functional decline of the organism. One of the key factors involved in this process is genomic instability and dysregulation of gene expression, which can result from alterations in the regulatory mechanisms that govern these processes^[1]^. Genetic alterations that contribute to the aging process include excessive DNA damage, loss of repair efficiency, and telomere attrition^[2,3]^, which can impact aging by causing genomic instability, influencing gene expression, and disrupting cellular functions. On the same line, epigenetic modifications over time affect the aging process as well, including chromatin remodeling, histone modifications, DNA methylation, and non-coding RNAs^[1,4]^. These genetic and epigenetic alterations are also likely to have consequences for miRNA biogenesis and miRNA-mediated gene regulation, as they can induce altered expression of specific miRNAs and miRNA biogenesis factors. miRNAs are small non-coding RNAs that play a crucial role in regulating gene expression at the post-transcriptional level, thanks to their ability to bind distinct mRNAs present in the cytoplasm. While miRNAs were initially thought to function primarily within the cell in which they were transcribed, we now know that they can also be secreted by cells and play an important role as extracellular signaling molecules in intercellular communication, partly via extracellular vesicles (EVs).

Many researchers have studied the changes in the expression of miRNAs, across the aging process, both in different tissues and in EVs^[5-10]^. A recent study explored the correlation between age and microRNA expression, obtaining a “miRNA age” from a linear model^[11]^. Based on this differential expression pattern, several miRNAs have been proposed as key molecules involved in the aging process, as many specific miRNAs have been shown to modulate aging-related pathways and traits^[12,13]^. Some of them have demonstrated effects on the lifespan of model organisms^[14,15]^.

Overall, when comparing cells or tissues from young and old individuals, differentially expressed miRNAs tend to be underexpressed in the latter^[16]^. This observation suggests that the molecular pathway that regulates miRNA biogenesis is altered during aging. The function of the factors involved in this pathway, which plays a crucial role in regulating gene expression, is impaired during aging in several tissues^[17,18]^. In addition, we previously showed that the miRNA biogenesis pathway is maintained in long-lived individuals, i.e., centenarians^[19,20]^. Through a comprehensive examination of the alterations of steps involved in miRNA biogenesis during aging, and its relationship with aging-related cellular and molecular traits, this review aims to provide valuable insights into the molecular mechanisms underlying aging and age-related diseases. We will also discuss how these alterations could explain the aberrant expression of different miRNAs in aged individuals, both in tissues and circulating miRNAs, ultimately contributing to our understanding of the aging process and potential interventions for promoting healthy aging.

## PRI-MIRNA TRANSCRIPTION AND AGING

The biogenesis of miRNAs initiates with the transcription of pri-miRNAs. pri-miRNAs are long precursor RNA molecules that serve as templates to produce miRNAs. The pri-miRNA is transcribed from the miRNA gene in the DNA by RNA polymerase II and is typically several hundred nucleotides long^[21]^. pri-miRNAs usually contain the transcripts of several miRNAs, which are termed co-transcribed miRNAs.

Aging is characterized by an accumulation of DNA damage; these DNA lesions can produce transcription blocks and accumulate during aging^[22-24]^. Recently, a group of researchers has demonstrated that almost half of elongating RNA polymerases are stalled in aged tissues, lowering productive transcription during aging^[25]^. Both DNA damage and RNA polymerase stalling may contribute to alterations in the transcription of pri-miRNAs during aging, as the accumulation of damage in genomic pri-miRNA regions could lead to changes in the function or a decrease in the expression of the mature miRNAs.

Interestingly, a recent study has shown that RNA polymerase II elongation speed increases with age in several species and during cellular senescence^[26]^. Authors observed that aging was associated with elevated numbers of mismatches with genome sequences, probably contributing to age-associated phenotypes. They also found that aged tissues have an increased formation of circular RNAs (circRNAs), which are miRNA post-transcriptional regulators, but not accumulation of miRNAs. This increase in circRNAs has been previously linked to an increased elongation speed of RNA polymerase II^[27]^. circRNAs and long non-coding RNAs (lncRNAs) are miRNA post-transcriptional regulators as they are thought to act as sponges^[28,29]^, binding to miRNAs and therefore repressing their function. Although the increase in elongation speed could, in theory, increase the transcription of miRNAs during aging, global miRNA levels are not usually increased with age^[5]^. This may be due to the alteration of subsequent steps in the miRNA biogenesis pathway and changes in post-transcriptional regulators of miRNAs, such as circRNAs and lncRNAs.

The dysregulation of pri-miRNA transcription has been mainly linked to the development of several types of cancer, whose incidence increases with age. Moreover, like cancer, there are genomic variations that can affect pri-miRNA transcription in aging, as well as epigenetic modifications of histones and DNA^[4]^. These epigenetic changes are highly associated with the aging process, for example, global hypomethylation and local hypermethylated sites shape the genome during aging^[30]^. Furthermore, during aging, there is a general loss of heterochromatin and detachment of lamina-associated domain (LAD) structures from the nuclear lamina. These changes in higher-order chromatin structure are accompanied by a redistribution of various histone modifications, such as histone methylation, acetylation^[4]^, and ubiquitylation^[31]^. Although there is scarce information on the epigenetic modifications that affect pri-miRNA regions during aging, a study with human samples linked the differences in methylation of miR-885 to successful cognitive aging^[32]^, highlighting the need for more research on the potential contribution of epigenetic changes to the pri-miRNA transcription alterations in aging.

In addition to genomic alterations, changes in the expression of transcriptional activators or repressors can also affect pri-miRNA transcription during aging. During cellular senescence, several transcription factors are responsible for driving the expression of senescence-associated pri-miRNAs, contributing to cellular senescence and tumor suppression^[33]^. For example, p53, a key player in the aging process and the maintenance of genomic integrity^[34]^, drives the expression of the miR-34 family. miR-34 has an important role in the development and maintenance of cellular senescence and dysregulation of the p53 pathway has been linked to age-related diseases, such as cancer, cardiovascular disease, and neurodegeneration^[35,36]^. c-Myc, a proto-oncogenic TF, downregulates several miRNAs, including miR-15a, miR-16-a, miR-34a, and let-7, which can help cells evade senescence^[37,38]^. Conversely, E2F1, part of the E2F family, displays a dual role in senescence by regulating both oncomiRs and tumor-suppressive miRNAs^[39]^. These TFs play important roles in modulating miRNA expression levels, contributing to cellular senescence and tumor suppression during aging^[33]^.

**Figure 1:**
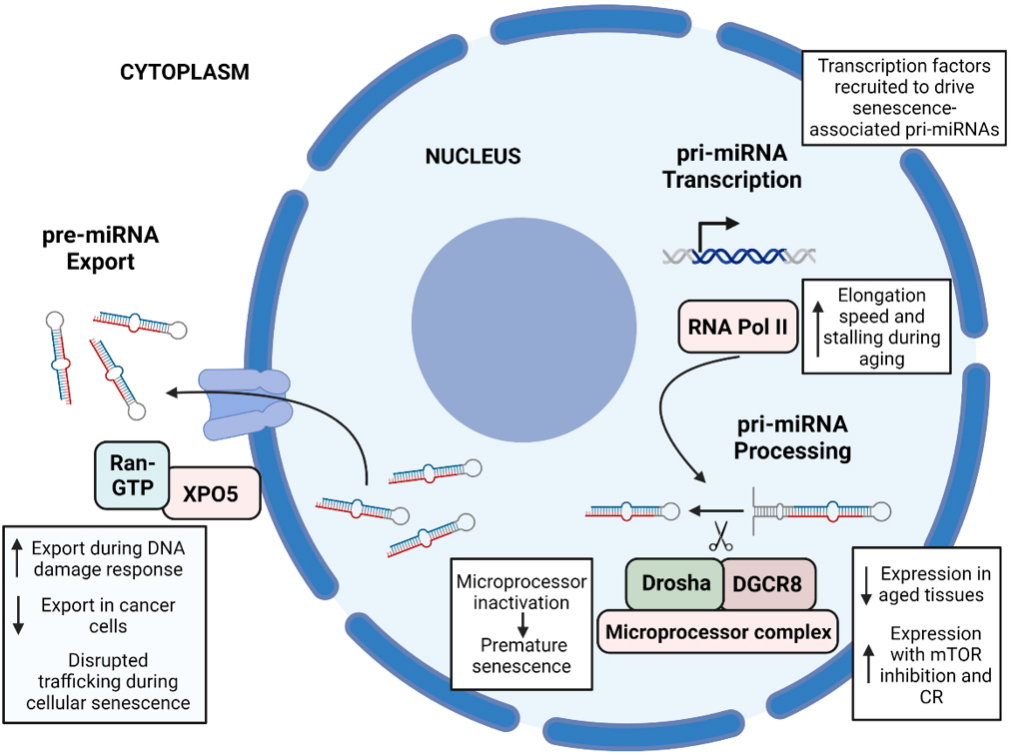
Summary of the main changes associated with aging in the first three steps of the miRNA biogenesis pathway. (1) pri-miRNA transcription: during aging, RNA Pol II, the enzyme that transcribes pri-miRNAs, has an increased elongation speed and stalling, leading to an increased incidence of transcription errors. During cellular senescence, several transcription factors such as p53 are recruited and drive the expression of senescence-associated pri-miRNAs. (2) pri-miRNA processing: some members of the microprocessor complex, mainly Drosha and DGCR8, show decreased expression and activity during aging, and its expression is enhanced by mTOR inhibition and caloric restriction. Microprocessor inactivation leads to premature senescence and proliferation defects. (3) pre-miRNA export: an increased export of miRNAs to the cytoplasm has been observed during DNA damage response, contrary to what happens in cancer cells, with a decreased export. Disrupted nucleocytoplasmic trafficking has been observed during cellular senescence. The figure was created using BioRender.

Overall, these changes could contribute to the alteration of the pri-miRNA transcription step during aging, which ultimately results in the aberrant expression of downstream target mRNAs that contribute to age-related pathologies [Figure 1].

## MICROPROCESSOR COMPLEX ALTERATIONS IN AGING

The second step in the biogenesis of miRNAs is the processing of pri-miRNAs into pre-miRNAs, which are ~ 70 nucleotide-long hairpin structures. The cellular component that operates this step is the microprocessor complex, which is composed of the proteins DGCR8 and Drosha^[40]^. The complex recognizes and cleaves the pri-miRNA at a specific position, known as the Drosha cleavage site, to generate the pre-miRNA^[41]^. The complex's activity is regulated by various factors. Negative feedback involves the cleavage and destabilization of DGCR8 mRNA mediated by the microprocessor. This mechanism helps to regulate the expression level of DGCR8 and maintains homeostatic control over miRNA biogenesis^[42,43]^.

The microprocessor complex is essential for the proper biogenesis of miRNAs. Loss of Drosha or DGCR8 in animal models leads to embryonic lethality, highlighting the critical role of the microprocessor complex in development. In addition, alterations in the microprocessor complex's activity have been implicated in various human diseases, including cancer and neurodegenerative disorders^[44]^.

In the aging field, researchers have looked at the expression levels of Drosha and DGCR8 during physiological aging, and studies have shown that Drosha and DGCR8 expression declines with age in some tissues^[17,19,45]^, while others have not found this decrease during physiological aging^[46,47]^. This reduction of the microprocessor activity has been directly linked to aging in *C. Elegans*, where the loss of microprocessor activity in the adult reduces lifespan and induces rapid aging^[48]^. For instance, reduced activity of Drosha and DGCR8 during physiological aging could lead to a decline in miRNA processing efficiency. This decline may lead to decreased production of specific miRNAs that are crucial for regulating aging-related pathways and processes. Some of the interventions with a demonstrated beneficial effect in aging, such as caloric restriction and inhibition of mTOR, have been shown to increase Drosha expression and miRNA biogenesis, suggesting a relationship between microprocessor activity and regulation of aging-related pathways and processes^[18]^.

These proteins are important for age-related processes such as cellular senescence. A recent study found that DGCR8 also plays a vital role in preserving heterochromatin organization^[49]^. An N-terminal-truncated form of DGCR8 caused premature senescence in human mesenchymal stem cells (hMSCs), irrespective of its microRNA-processing function. DGCR8 was downregulated in naturally and pathologically aged hMSCs, whereas its overexpression reversed the senescent phenotype and improved mouse osteoarthritis^[49]^. Indeed, the inactivation of DGCR8 results in an antiproliferative response and acquisition of a senescent phenotype accompanied by the upregulation of the cell-cycle inhibitor p21 in fibroblasts^[50]^. In human MSCs, knockdown of DGCR8 expression induced significant proliferation defects and exhibited senescence-associated changes, such as elevated levels of reactive oxygen species (ROS) and decreased levels of the antioxidant enzyme superoxide dismutase 2^[51]^.

Microprocessor complex dysregulation contributes to the age-related changes in miRNA expression and has been proposed as a contributor to the development of age-related diseases. For example, in Alzheimer's disease (AD), a recent study found that levels of Drosha protein were significantly lower in neurons of the cortex and hippocampus. They demonstrated that amyloid-beta oligomers, a hallmark of AD, led to the phosphorylation of Drosha and its redistribution from the nucleus to the cytoplasm, causing a decrease in its level. Furthermore, overexpressing Drosha protected neurons from Aβ oligomers-induced apoptosis^[47]^. Regarding Parkinson’s disease, authors discovered that the level of Drosha decreases in cellular and animal models of Parkinson’s, partially mediated by the phosphorylation of p38 MAPK. Increasing the level of Drosha protected dopamine-producing neurons from 6-OHDA-induced toxicity, both *in vitro* and *in vivo*, and improved the motor deficits of these mice^[52]^. DGCR8 has also been linked to heart failure, a common age-related condition. Cardiomyocyte-specific deletion of DGCR8 resulted in left ventricular malfunction, dilated cardiomyopathy, and premature lethality, indicating the critical role of the miRNA biogenesis pathway in maintaining cardiac function^[53]^.

Therefore, alterations of the microprocessor complex, which plays a crucial role in the biogenesis of miRNAs, have been linked to several age-related diseases. This complex may be implicated in some aging-related processes such as cellular senescence or oxidative stress and is regulated by caloric restriction [Figure 1].

## PRE-MIRNA EXPORT AND AGING

The export of pre-miRNAs is an essential step in the biogenesis of miRNAs, and it is the process by which pre-miRNAs are transported from the nucleus to the cytoplasm, where they are further processed into mature miRNAs. This process is mediated by the Exportin-5 protein (XPO5), which recognizes and binds to pre-miRNAs in a complex with the nuclear Drosha. The XPO5-pre-miRNA complex is then transported through the nuclear pore complex and released into the cytoplasm^[54]^. The small GTPase Ran is the other component of the pre-miRNA export machinery. In its GTP-bound state, Ran binds to the nuclear pore complex and facilitates the translocation of RNA-binding proteins (RBPs) and RNA molecules between the nucleus and cytoplasm^[55]^. These RBPs, which are important factors for the export of pre-miRNAs, have been proposed as modulators of aging, as they are involved in the decoupling of mRNA/protein expression of the brain that characterizes aging^[56]^.

Little is known about the specific role of miRNA export alterations in the aging process; however, it has been linked to some mechanisms associated with aging. For example, an accelerated export of pre-miRNAs has been shown after DNA damage in the ataxia telangiectasia mutated (ATM)-dependent manner^[57]^, suggesting that an increased release of pre-miRNA to the cytoplasm may be part of the DNA damage response, which is tightly linked to genomic stability and aging. ATM kinase, a core component of DNA damage response, has been linked to cell senescence, stem cell dysfunction, and aging^[58]^. Interestingly, in cancer cells, the miRNA export modulation seems to be the opposite, as XPO5 genetic defect traps pre-miRNAs in the nucleus, reduces miRNA processing, and diminishes miRNA-target inhibition in cancerous cells. The restoration of XPO5 functions reverses the impaired export of pre-miRNAs and has tumor-suppressor features^[59]^.

Ran GTPase-regulated nucleocytoplasmic transport plays an important role in DNA damage response and cell cycle. Increased RanGTP levels lead to an accelerated cell cycle and enhanced DNA damage repair^[60]^. Furthermore, cells with overexpression of RanGTP can evade DNA damage-induced senescence. These findings indicate that changes in the activity of RanGTP-regulated nucleocytoplasmic transport can modulate the cell cycle rate and the efficiency of DNA repair^[61]^.

During cellular senescence, a dysregulation of nucleocytoplasmic trafficking has been described^[62]^. A recent study analyzing the proteins and transcripts in young and senescent cells found that nucleocytoplasmic trafficking disruption is a key feature of replicative senescence. Blocking this process induced a senescence-like phenotype, suggesting an important role of trafficking in the nuclear membrane in senescence^[63]^. Although nucleocytoplasmic trafficking is a fundamental process with relevance extending beyond miRNA trafficking, alterations in the exchange of miRNAs between the nucleus and the cytoplasm may have a role in cellular senescence.

Regarding specific diseases, there is still little evidence of a link between alterations in miRNA export machinery and the development of age-related diseases; however, some studies have shown that disruption of nucleocytoplasmic transport occurs in AD, contributing to tau and amyloid-β neurotoxicity^[64,65]^.

Regulation of the trafficking of different molecules, such as miRNAs, from the nucleus to the cytoplasm, is then a noteworthy factor influencing processes linked to aging. More specifically, DNA damage response and cellular senescence are characterized by an alteration in this trafficking, making it an attractive target for future studies regarding the molecular traits associated with aging [Figure 1].

## PRE-MIRNA PROCESSING ALTERATIONS IN AGING

Pre-miRNA processing is the last step in the miRNA biogenesis pathway. Upon reaching the cytoplasm, the pre-miRNA encounters the enzyme Dicer, an RNase III enzyme. Dicer recognizes the hairpin structure of the pre-miRNA and cleaves it near the terminal loop region^[66]^. This cleavage event generates a short RNA duplex consisting of two complementary strands: the guide strand (mature miRNA) and the passenger strand. The less stable passenger strand is typically degraded, while the guide strand remains associated with the RNA-induced silencing complex (RISC). The RISC complex consists of a mature miRNA and several proteins, including Argonaute (AGO) and GW182. The RISC complex recognizes target mRNAs through base pairing interactions between the miRNA and the mRNA's 3' untranslated region (UTR). Then, it binds to the target mRNA, leading to mRNA degradation or translational repression^[67,68]^. The interplay between Dicer and Argonaute proteins in the miRNA pathway is a crucial aspect of miRNA biogenesis, with both proteins playing complementary roles to ensure the proper processing and function of mature miRNAs.

## AGE-RELATED CHANGES IN DICER PROTEIN

Among all the factors that are implicated in miRNA biogenesis, the most studied regarding the aging field is probably Dicer^[69]^. For instance, decreased expression of Dicer has been reported in aged tissues of various organisms, including humans, mice, and fruit flies^[70-72]^.

Excess adipose tissue is linked to metabolic disease and reduced lifespan, while caloric restriction (CR) reduces these risks^[73-75]^. Mori *et al*. were one of the first to study the specific role of Dicer and miRNA processing in adipose tissue during aging^[17]^. They observed a decline in Dicer expression and miRNA levels in aging mice and in *C. elegans*, which was prevented by CR. Similar reductions in Dicer expression were found in preadipocytes from elderly humans. Knockdown of Dicer in cells led to premature senescence, and mice with fat-specific Dicer knockout exhibited increased sensitivity to oxidative stress. In *C. elegans*, loss-of-function mutations in Dicer decreased lifespan and stress tolerance, while overexpression of Dicer in the intestine improved stress resistance^[17]^. Using Dicer1 deficient mice, authors recently showed that these mice develop premature aging phenotypes in several tissues, including skin, heart, and adipose tissue^[76]^.

On the same line, fat-specific Dicer knockout mice (AdicerKO) were used to investigate the role of adipose tissue Dicer in the metabolic effects of aging and CR. AdicerKO mice showed reduced oxidative metabolism, increased lactate in adipose tissue, and structural and functional changes in mitochondria. They exhibited increased mTORC1 activation in adipose tissue and skeletal muscle, leading to accelerated age-associated insulin resistance and premature mortality. Furthermore, the insulin-sensitizing effects of CR were abolished in AdicerKO mice^[71]^.

Another study linked Dicer to the metabolic adaptations induced by aerobic exercise training. This study showed that aerobic exercise training leads to the upregulation of Dicer in the adipose tissue of both mice and humans. The researchers also found that this upregulation can be mimicked by infusing serum from exercised mice into sedentary mice, indicating the involvement of circulating factors. Adipocyte-specific Dicer is essential for the metabolic adaptations induced by exercise training, as it enables controlled substrate utilization in adipose tissue, which in turn supports skeletal muscle function. Exercise training also results in an overall increase in miRNA expression in adipose tissue^[70]^

As shown, miRNAs play a critical role in regulating gene expression and are implicated in neuronal development, aging, and neurodegenerative diseases such as Parkinson's disease^[77]^. In this regard, Dicer has been shown to regulate the functional maintenance of adult dopamine (DA) neurons. Researchers observed reduced Dicer levels in the ventral midbrain and altered miRNA expression profiles in aged mice DA neurons. By selectively removing Dicer in adult mice DA neurons, they found decreased levels of striatal dopamine and its metabolites. Dicer KO mice experienced progressive loss of DA neurons, leading to severe locomotor deficits. Additionally, they discovered that pharmacological stimulation of miRNA production promoted the survival of cultured DA neurons and reduced their vulnerability to endoplasmic reticulum stress^[78]^. Regarding neurodegeneration, it is known that impairment of angiogenesis contributes to this process. Researchers observed that Dicer1 expression decreased with age both in cerebromicrovascular endothelial cells and small cerebral vessels in rats. Aged cells showed altered miRNA expression profiles, leading to impaired proliferation, adhesion, migration, and the formation of capillary-like structures. Overexpression of Dicer1 in these cells partially restored the miRNA profile and improved angiogenic processes. Conversely, the downregulation of Dicer1 in young cells resulted in a similar miRNA profile and impaired angiogenic functions^[79]^.

Not only does the expression level of Dicer seem to be important for its function. Interestingly, diurnal oscillations of Dicer expression have been found in key clock control systems such as the suprachiasmatic nucleus, retina, liver, and bone marrow. However, these oscillations were reduced or shifted in aging and diabetes. The decrease and shift of Dicer expression were accompanied by similar changes in miRNAs 146a and 125a-5p, as well as an increase in toxic Alu RNA^[80]^.

Moreover, phosphorylated Dicer present in the nucleus has a key role in the response to DNA double-strand breaks^[81]^. In a recent study in mice, researchers studied constitutive phosphorylation in Dicer1. Mice with a phosphomimetic mutation in Dicer1 exhibited high postnatal lethality, accelerated aging, and infertility in the surviving individuals. The introduction of dual-phosphomimetic mutations exacerbated these defects and led to alterations in metabolism-related miRNAs and a hypermetabolic phenotype^[82]^.

Mechanistically, Dicer influences various hallmarks of aging, including cellular senescence, oxidative stress, or genomic instability. Reduced Dicer expression leads to increased oxidative stress and premature senescence^[17]^. Additionally, phosphorylated Dicer has been linked to the cellular response to DNA damage^[81]^, suggesting a potential role in maintaining genomic stability. While further investigations are needed, the existing body of evidence points to Dicer as a player connecting miRNA biogenesis to multiple hallmarks of aging. All these results suggest that regulation of miRNA processing by Dicer plays an important role in the aging process and longevity; therefore, modifications in Dicer expression could have a significant role in the treatment of age-related diseases.

## ARGONAUTE PROTEIN FAMILY IN AGING AND LONGEVITY

The Argonaute protein family is an essential component of the RISC, and alterations in its functions have been associated with aging and longevity, although its role in aging is not as established as that of Dicer. Argonaute proteins have been shown to participate in aging-related processes, such as senescence^[83]^ and proteostasis^[84]^.

Loss of pre-miRNA maturation in adulthood results in a shortened lifespan in *C. elegans*; more specifically, adult-specific loss of argonaute-like gene-1 (ALG-1) activity results in accelerated aging^[85]^. In adul*C. elegans*, ALG-1 knockdown leads to a shortened lifespan, whereas ALG-2 knockdown in nematodes increases lifespan^[86]^. Analysis of gene expression in adult worms revealed that different sets of miRNAs and protein-coding genes are misregulated in ALG-1 and ALG-2 mutants. Authors propose that distinct modulation of the Insulin/IGF-1 signaling pathway by downstream factors could partially explain these differences in lifespan. However, it remains unclear whether the contrasting roles of ALG-1 and ALG-2 during aging are due to differences in expression or protein function^[86]^.

In Drosophila, recent work has studied the effect of the knockdown of Argonaute genes (AGO1, AGO2, AGO3, and piwi) in different tissues. The downregulation of these genes resulted in reduced or insignificant changes in lifespan and survival after γ-irradiation. However, the specific knockdown of piwi in the fat body and nervous system increased lifespan, suggesting a specific role of these proteins in aging depending on the expressed tissue^[87]^. During normal aging, the activity of transposable elements increases significantly in the brain^[88,89]^. Mutations in the AGO2 protein of Drosophila led to even higher expression of transposons in the brain, leading to progressive memory decline and a reduced lifespan that worsened with age^[90]^. These findings connect Argonaute with the activation of transposable elements during aging.

During cytokine-induced senescence, AGO2 was found to translocate from the cytoplasm to the nucleus of senescent cells in a reversible manner, suggesting that AGO2 contributes to stable cell cycle arrest and regulation of growth in cytokine-induced senescence^[83]^. Argonaute proteins, along with miR-9, have been shown to regulate the balance between neural stem cells' quiescence and activation in zebrafish, which is important for maintaining adult germinal pools^[91]^. In *C. elegans,* ALG-1 and ALG-2 help to maintain proteostasis, specifically in ubiquitin-dependent degradation pathways such as ubiquitin fusion degradation and endoplasmic reticulum-associated protein degradation^[84]^.

**Figure 2:**
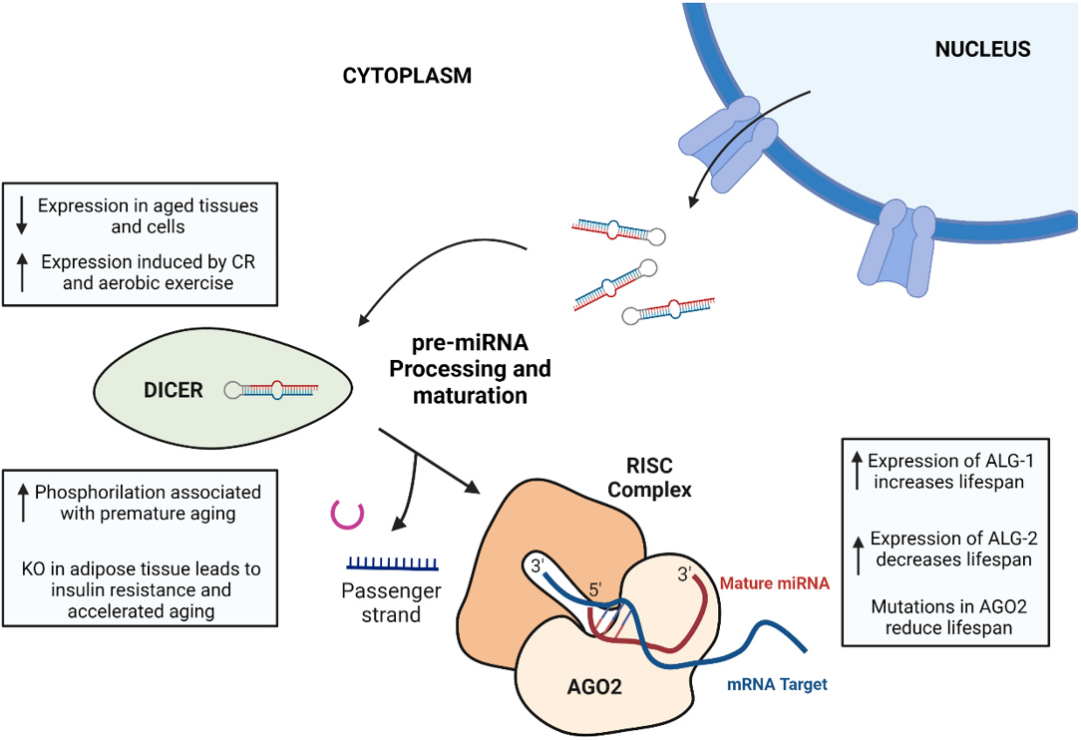
Summary of the main changes associated with aging in the last steps of miRNA biogenesis pathway: pre-miRNA processing and maturation. (1) Dicer expression is generally reduced in aged tissues and cells, whereas its expression is induced by caloric restriction and aerobic exercise. In mice, a phosphomimetic mutation in Dicer1 induces accelerated aging, and specific KO of Dicer in adipose tissue has important effects on metabolism, leading to insulin resistance and premature aging. (2) The role of Argonaute proteins in aging is not well-established, as ALG-1 and ALG-2 (two Argonaute proteins in *C. Elegans*) have contrary effects on the lifespan of nematodes, and AGO2 mutations in Drosophila cause a reduced lifespan. The figure was created using BioRender.

Therefore, the dysregulation of the machinery involved in pre-miRNA processing emerges as an important aspect of miRNA-mediated gene regulation in aging, contributing to age-related phenotypes and diseases [Figure 2].

## CONSEQUENCES OF BIOGENESIS ALTERATIONS IN CIRCULATING MIRNAS

miRNAs not only exert their functions in the cells of origin, but also can be secreted in the extracellular environment and reach distant cells and tissues. miRNAs are usually secreted along with different binding proteins, such as AGO2 and lipoproteins^[92]^. In the last decade, the study of EVs and their components has suggested that miRNAs are important cargoes of EVs^[93]^. The biological relevance of intercellular communication through miRNAs present in EVs is still under debate^[94-96]^. However, there is a growing body of evidence that confirms the fact that miRNA content in EVs is altered during several conditions, whereas pathological or physiological^[6,97-99]^.

Therefore, it is reasonable to think that the alterations of the miRNA biogenesis pathway during aging could lead to an altered content in circulating miRNAs. This assumption has been validated by multiple research works that have looked into the differential expression of miRNAs across different ages in the circulation [Table 1], showing that miRNA profile changes with age in humans and identifying specific miRNAs with differential expression in aged individuals. A recent study investigated the associations between age and whole-blood microRNA expression, and researchers identified 127 microRNAs that were differentially expressed with age, with most of them being underexpressed in older individuals, and developed a linear model to predict age. The difference between microRNA age and chronological age was associated with all-cause mortality^[11]^.

**Table 1:** Outline of the top circulating miRNAs, target pathways, and the effect of aging on their expression

**miRNA**	**Main target pathways (GeneCards)**	**Effect of aging on its expression**	**Sample used**	**Reference**
miR-21	TGF-β Immune response	Increase (decreased in centenarians)	Human plasma	[102]
miR-151a-5p	Inflammation	Decrease	Human serum	[103]
miR-181a-5p	Inflammation			
miR-1248	Inflammation			
miR-378	IGFR1 AKT	Increase	Human serum and plasma	[104]
miR-20a-3p	Cell differentiation DNA damage response	Decrease		
miR-30b-5p	Extracellular matrix			
miR-106b-5p	Immune response DNA damage response			
miR-191	Cell cycle regulation			
miR-301a	Smooth muscle cell proliferation			
miR-374a	Cell adhesion			
miR-206	Cell differentiation	Increase	Human serum	[105]
miR-486	Cell differentiation			
miR-19a-3p	Immune response DNA damage response EVs intercellular communication	Decrease		
miR-10b-5p	Lipid storage EGFR signaling	Increase	Human plasma	[16]
miR-193a-5p	DNA damage response Angiogenesis			
miR-374a-5p	miRNA-mediated gene silencing Cell adhesion	Decrease		
miR-26b-5p	Inflammation			
miR-191-5p	Cell cycle regulation			
miR-30e-3p	Cardiomyocyte hypertrophy			
miR-26a-5p	Cell differentiation			
miR-9-3p	Immune response Cell differentiation	Increase	Human serum and plasma	[106]
miR-206	Cell differentiation	Decrease		
miR-129-2-3p	Fatty acid biosynthesis MAPK signaling			
miR-203a-3p	Immune response DNA damage response			
miR-11181-3p	Wnt signaling			

The table was generated with human studies that used serum or plasma samples to extract the RNA for profiling (i.e., not whole-blood, cell, or tissue-specific miRNAs).

Along with miRNAs, EVs can also carry miRNA biogenesis regulators, modulating miRNA production and processing in recipient cells. EVs have been shown to carry important proteins for miRNA biogenesis, such as Dicer or AGO2^[100,101]^; they can also carry miRNAs that target the miRNA biogenesis pathway. Understanding how miRNA biogenesis regulators are transported via EVs and how they impact miRNA production in recipient cells is important to consider when investigating miRNA alterations during aging.

Although the circulating miRNA expression profile could be affected by innumerable cellular processes and pathways, we hypothesize that the dysregulation of the miRNA biogenesis pathway during aging can lead to alterations in circulating miRNAs. This could contribute to the altered intercellular communication seen during aging, as impairment of the miRNA biogenesis pathway can have deleterious effects in the intracellular environment and distant cells. Further research is needed to uncover the relevance of miRNA biogenesis changes during aging in the differential expression of circulating miRNAs.

## CONCLUSION

The dysregulation of miRNA biogenesis at various steps, including pri-miRNA transcription, microprocessor-complex activity, pre-miRNA export, and pre-miRNA processing, has been implicated in the aging process. This can result from genetic alterations, epigenetic modifications, and changes in the expression of regulatory factors. Thus, miRNA biogenesis alteration can lead to aberrant expression of downstream target mRNAs, contributing to age-related pathologies.

Studies on pri-miRNA transcription during aging are limited, but evidence suggests that genomic alterations and changes in the expression of transcriptional activators or repressors can affect this step^[25,35,36]^. The dysregulation of the microprocessor complex, composed of DGCR8 and Drosha, has been linked to age-related diseases and cellular senescence^[49]^. The expression levels of Drosha and DGCR8 during physiological aging seem to decrease, but more importantly, interventions such as caloric restriction and inhibition of mTOR have been shown to increase their expression and miRNA biogenesis^[18]^. The specific role of pre-miRNA export from the nucleus to the cytoplasm in aging is not well understood. However, accelerated export of pre-miRNAs has been observed in response to DNA damage^[57]^, and changes in nucleocytoplasmic trafficking have also been implicated in cellular senescence^[63]^.

The final step of miRNA biogenesis, pre-miRNA processing, is the most studied in the aging field. Reduced expression of Dicer has been observed in aged tissues of various organisms^[70-72]^. Studies have shown that decreased Dicer expression in adipose tissue is linked to metabolic disease and reduced lifespan, while caloric restriction can prevent these effects^[17,71]^. Additionally, Dicer expression has been implicated in the metabolic effects of aging, insulin resistance, and the adaptations induced by aerobic exercise training^[70,71]^. Argonaute protein alterations have also been associated with lifespan and senescence^[83,86]^.

Overall, the dysregulation of miRNA biogenesis contributes to the development of age-related diseases and processes such as cellular senescence and genomic instability. Further research is needed to better understand the specific mechanisms underlying these impairments and their implications in aging. Targeting the miRNA biogenesis pathway holds potential for therapeutic interventions to mitigate age-related pathologies and promote healthy aging.

## DECLARATIONS

### Copyright

© The Author(s) 2023.
